# For Better or for Worse: A Look Into Neutrophils in Traumatic Spinal Cord Injury

**DOI:** 10.3389/fncel.2021.648076

**Published:** 2021-04-22

**Authors:** Sandra Zivkovic, Maryam Ayazi, Grace Hammel, Yi Ren

**Affiliations:** Department of Biomedical Sciences, Florida State University College of Medicine, Tallahassee, FL, United States

**Keywords:** spinal cord injury, neutrophils, secondary injury, inflammation, cytokines, myelin debris

## Abstract

Neutrophils are short-lived cells of the innate immune system and the first line of defense at the site of an infection and tissue injury. Pattern recognition receptors on neutrophils recognize pathogen-associated molecular patterns or danger-associated molecular patterns, which recruit them to the destined site. Neutrophils are professional phagocytes with efficient granular constituents that aid in the neutralization of pathogens. In addition to phagocytosis and degranulation, neutrophils are proficient in creating neutrophil extracellular traps (NETs) that immobilize pathogens to prevent their spread. Because of the cytotoxicity of the associated granular proteins within NETs, the microbes can be directly killed once immobilized by the NETs. The role of neutrophils in infection is well studied; however, there is less emphasis placed on the role of neutrophils in tissue injury, such as traumatic spinal cord injury. Upon the initial mechanical injury, the innate immune system is activated in response to the molecules produced by the resident cells of the injured spinal cord initiating the inflammatory cascade. This review provides an overview of the essential role of neutrophils and explores the contribution of neutrophils to the pathologic changes in the injured spinal cord.

## Introduction

Innate immunity is the first line of defense against foreign agents and self-tissue injury (Buchmann, 2014; Hato and Dagher, 2015). The innate response is much faster than adaptive immunity and can be initiated immediately or within a few hours (Hato and Dagher, 2015). The innate immune response results in inflammation to control the infection or injury and signal the recruitment of relevant immune cells, which aid in clearing the pathogens and cell debris while promoting tissue healing and recovery (Newton and Dixit, 2012; Cui et al., 2014; Spiering, 2015). The components of the innate immune system that aid in its function are granulocytes, monocytes, natural killer cells, and the complement system (Spiering, 2015). Neutrophils, also known as polymorphonuclear leukocytes, are the key players of the innate immune system and the first immune cells to arrive at the site of infection and injury (Kobayashi and DeLeo, 2009; Rosales et al., 2017; Kovtun et al., 2018). In humans, neutrophils are produced at a rate of 1 × 10^11^ cells per day and are the most abundant granulocytes, comprising 60–70% of all blood leukocytes and have a short life span of fewer than 24 h in the bloodstream (Hong et al., 2012; Mayadas et al., 2014; McCracken and Allen, 2014; Sheshachalam et al., 2014). In mice, neutrophils are the most common granulocytes and are produced at a rate of 1 × 10^7^ cell per day, comprising 20–30% of all blood leukocytes (O’Connell et al., 2015; Ng et al., 2019). Mature circulating neutrophils are destined for apoptosis and clearance by macrophages (Mϕ) in the liver, spleen, and bone marrow to maintain homeostasis (Savill et al., 1989; Fox et al., 2010; Hong et al., 2012; Greenlee-Wacker, 2016). This review describes the involvement of neutrophils in different pathological states with a focus on spinal cord injury (SCI).

SCI is a traumatic and detrimental condition that can result in temporary or permanent paralysis in injured patients (Kumar et al., 2018). An estimated 700,000 new SCI cases arise per year worldwide, resulting in a global incidence of 10 cases per 100,000 people (Jin et al., 2019). The vast majority of SCI cases are traumatic and caused by accidents in traffic, sports, falls, and violence (Alizadeh et al., 2019). The major phases of injury response after SCI can be categorized into the primary phase and secondary phase of injury (Ahuja et al., 2017; Alizadeh et al., 2019). Immediately after an SCI, the resulting initial mechanical damage, commonly referred to as primary injury, is characterized by a mechanical force acting on the spinal cord, resulting in immediate hemorrhage, cell death, vascular damage, ischemia, tissue disruption, edema, and the physical disruption of neurons at the site of injury (Oyinbo, 2011; Singh et al., 2012). The primary phase initiates a series of molecular changes at the tissue and cellular levels contributing to the secondary injury cascade, resulting in further permanent damage and neurological dysfunction. Secondary injury can be further divided into the acute, the subacute, and the chronic subphases (Badhiwala et al., 2018).

### Inflammatory Response: A Call for Neutrophils

**FIGURE 1:**
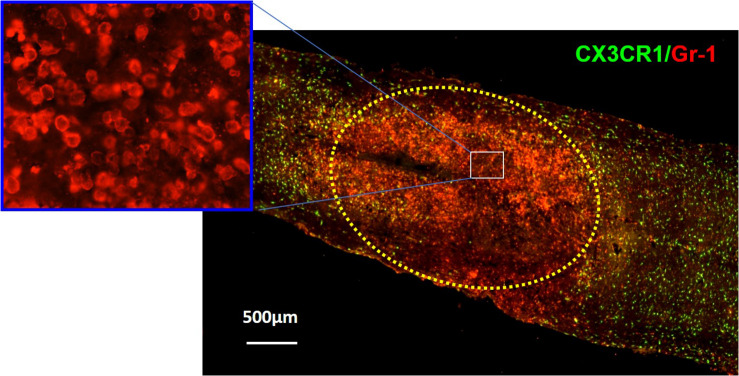
Immunohistochemical analysis showing neutrophils (red) in the injury site at 24 h after SCI in CX3CR1^GFP/+^ mice where CX3CR1 is a marker for microglia, and Gr-1 is a marker for neutrophils. The injury lesion is circled with dotted lines. Although neutrophils can be detected as early as 3 h postinjury and peak 12 h postinjury, they remain elevated up to 24 h postinjury and decrease in concentration 3 days post-SCI. Adapted from Wang et al. (2015).

The first cells to be recruited to the injury site are neutrophils (Figure 1; Popovich and Jones, 2003; Oyinbo, 2011; Kubota et al., 2012; Wang et al., 2015; Guo et al., 2016). To respond to a pathogenic invasion or tissue damage, pattern recognition receptors (PRRs) on neutrophils recognize pathogen-associated molecular patterns (PAMPs) or danger-associated molecular patterns (DAMPs) (Suresh and Mosser, 2013; Amarante-Mendes et al., 2018). The PRRs activate downstream signaling pathways such as the mitogen-activated protein kinase and nuclear factor κB (NF-κB) pathways responsible for upregulating proinflammatory cytokines and chemokines (Chen and Nunez, 2010; Kigerl et al., 2014; Venereau et al., 2015; Pouwels et al., 2017; Kaur et al., 2019). PAMPs, DAMPs, and their respective receptors on neutrophils are summarized in Table 1 (Parroche et al., 2007; Mogensen, 2009; Chen and Nunez, 2010; Kumar et al., 2011; Venereau et al., 2015; Roh and Sohn, 2018; Wang, 2018).

**TABLE 1:** Examples of PAMPs and DAMPs and their respective receptors on neutrophils.

PAMPs:	Receptors:	DAMPs:	Receptors:
Viral ssRNA dsRNA LPS Lipoarabinomannan Zymosan Lipoteichoic acid CpG motifs of bacteria and viruses Bacterial flagellin Triacyl lipoproteins Fungal mannose Parasitic hemozoin	TLR7/8 RIG-I, MDA5, PKR TLR4 TLR2 TLR9 TLR5 TLR1/TLR2 Mannose receptor, dectin-2, DC-SIGN TLR9	RNA DNA Histones HMGB1 S100 proteins Biglycan N-formyl peptides ATP Interleukin 1α Interleukin 33 Heat shock proteins (HSPs) Amyloid-β	TLR3, TLR7, TLR8, RIG-I, MDA5 TLR9, AIM2 TLR2, TLR4 TLR2, TLR4, RAGE TLR2, TLR4, RAGE TLR2, TLR4, NLRP3 FPR1 P2X7, P2Y2 IL-1R ST2 TLR2, TLR4, CD91 TLR2, NLRP1, NLRP3, CD36, RAGE

Tissue damage in SCI is first detected by resident cells in the spinal cord, such as glial cells and microvascular cells, resulting in proinflammatory chemokine expression that attracts neutrophils to the injured area (Anwar et al., 2016; Alizadeh et al., 2019). The most common proinflammatory cytokines and chemokines that facilitate neutrophil recruitment include interleukin 1α (IL-1α), IL-β, IL-8, tumor necrosis factor (TNF), granulocyte colony-stimulating factor, CCL3, CXCL1, CXCL2, and CXCL5 (Kobayashi et al., 2018; Pelisch et al., 2020). IL-1β is a crucial initial proinflammatory cytokine produced after injury. Within an hour after contusion SCI in mice, astrocytes and microglia release IL-1β, which peaks in expression 12 h postinjury, correlating with the peak of infiltrated neutrophils to the injured area (Pineau and Lacroix, 2007; Saiwai et al., 2010; Kubota et al., 2012). IL-1β binds IL-1R on the cells, activating the production of proinflammatory cytokines through the NF-κB pathway (Zhang and Fuller, 2000; Albrecht et al., 2007). Astrocytes produce two important neutrophil chemoattractants, CXCL1 and CXCL2 (Pineau et al., 2010). Deletion of IL-1R in mice showed a significant reduction in infiltration of neutrophils to the injured spinal cord (Pineau et al., 2010). Administration of IL-1 receptor antagonist (IL-1RA) inhibits IL-1 signaling and suppresses neutrophil infiltration in the injured spinal cord (Yates et al., 2021). SCI mice treated with colony-stimulating factor 1 receptor antagonist PLX5622 have significantly reduced neutrophil infiltration in the injured spinal cord (Li et al., 2020). Additionally, deletion of MyD88, an adapter molecule of IL-1R and an intermediate protein in the activation of the NF-κB pathway, showed low expression of CXCL2 and no expression of CXCL1, resulting in low recruitment of neutrophils in mouse SCI (Gasse et al., 2007; Pineau et al., 2010). IKK-β is a regulatory unit of the NF-κB pathway. When bound to NF-κB molecules, it keeps this pathway inactive as it prevents NF-κB molecules from translocating into the nucleus (Karin, 1999; Rothwarf and Karin, 1999; Hacker and Karin, 2006). Similarly to the deletion of Myd88, myeloid cell–specific IKK-β–deficient mice showed decreased CXCL1 expression in the injured spinal cord and less neutrophil recruitment, strengthening the importance of these chemoattractants for the neutrophil infiltration via the NF-κB pathway (Kang et al., 2011). Moreover, neutrophil infiltration into the injured spinal cord is also attributed to the tight regulation of the receptor for the complement activation product 3a, C3aR1. In C3aR1 knockout (KO) mice, CXCL1 level increases 2 h post-SCI and remains elevated after the injury, suggesting that C3aR1 negatively regulates neutrophil mobilization by acting as the antagonist for neutrophil chemotactic signals (Brennan et al., 2019).

## Major Functions of Neutrophils in the Injured Spinal Cord

The role of neutrophils at the injured spinal cord is not well understood. As mentioned earlier, upon SCI, there is physical damage to the tissue, which generates cell debris (Oyinbo, 2011; Singh et al., 2012). What is the exact role of neutrophils in response to the present debris in the injured area is yet to be elucidated. However, there are three major mechanisms by which the neutrophils generally respond to an inflammation and/or infection: degranulation, phagocytosis, and formation of neutrophil extracellular traps (NETs) (Rosales, 2020).

### Degranulation

Very little is known about the role of degranulation in the pathophysiology of SCI, but a valuable lesson can be learned from its general role in other diseases. Degranulation of neutrophils is when granules directly translocate and fuse with the plasma membrane and release their contents into the extracellular space (Lacy, 2006). Upon an extracellular stimulus, secretory vesicles are mobilized and regulate circulating neutrophil transformation to an activated state (Table 2) where they then secrete their granular contents into the extracellular space (Borregaard et al., 1990; Ramadass and Catz, 2016). The purpose of degranulation into the extracellular space is to kill the extracellular enemy (Yaseen et al., 2017). Degranulation is stimulated upon ligand binding, such as IL-8, to its G-protein–coupled receptor CXCR1/2 (Barlic et al., 2000). Translocation of the granules to the neutrophil plasma membrane depends on the actin remodeling and microtubule assembly (Burgoyne and Morgan, 2003). Additionally, it requires increase in intracellular Ca^2+^ concentration and hydrolysis of ATP and GTP (Lacy, 2006). Fusion of granules with the plasma membrane deposits granular component cytochrome b_558_ onto the plasma membrane and stimulates assembly of nicotinamide adenine dinucleotide phosphate (NADPH) and reactive oxygen species (ROS) production (McLeish et al., 2013). Rab-GTPase family regulates secretion of granular contents in a time-dependent and granule-specific manner (Ramadass and Catz, 2016). Neutrophils’ primary granules contain a spectrum of serine proteases listed in Table 3, which effectively kill the pathogen (Teng et al., 2017). Although these granular contents are potent weapons to kill pathogens, they are also toxic to the tissue (Kruger et al., 2015). As infiltrated neutrophils are accumulated in the demyelinating lesion core (Figure 1), where the area for new blood vessel formation occurs, further studies are needed to know the functional consequences of neutrophil degranulation in the pathogenesis of SCI, such as destruction of myelin sheath and breakdown of the blood–spinal cord barrier (BSCB).

**TABLE 2:** Common cytokines and chemokines expressed constitutively or upon activation of neutrophils.

Proinflammatory cytokines	Anti-inflammatory cytokines	Chemokines
IL-1α IL-1β IL-6 IL-7 IL-9 IL-16 IL-17 IL-18 TNF-α MIF	IL-4 IL-1 TGF-β1 TGF-β2	CCL2 CCL3 CCL4 CCL17 CCL18 CCL19 CCL20 CCL22 CXCL1– CXCL6, CXCL8–13

**TABLE 3:** Neutrophilic granules and function—primary, secondary, and tertiary.

Primary—azurophilic	Secondary—specific	Tertiary—gelatinase
Function: Contain potent hydrolytic enzymes that kill and digest microbes	Function: Help with the replenishment of membrane components and free radical reactions	Function: Help with the replenishment of membrane components and free radical reactions
Granule components: Cathepsin G Elastase Myeloperoxidase Azurocidin Defensins Acid hydrolases Lysozyme BPI Phospholipase A_2_ Proteinase 3 CD63 CREG1 Lysosome-associated membrane protein 2 (LAMP2) Complement C3	Granule components: Lactoferrin Cathelicidin, Collagenase Gelatinase B Cytochrome b_558_ Lysozyme IL-10R Calprotectin Secretory phospholipase Haptoglobin Neutrophil gelatinase-associated lipocalin (NGAL)	Granule components: Cathelicidin Collagenase Gelatinase B Cytochrome b_558_ IL-1RA TRAIL Heparanase BAFF MMP9

### Phagocytosis of Cell Debris

Phagocytosis is a cellular process for engulfing and eliminating self or nonself particles. Particles opsonized with immunoglobulins (Ig), IgG or IgM, and complement factors are phagocytosed more effectively via Fcγ receptors and complement receptors (CRs) on neutrophils, respectively (Flannagan et al., 2009; Yu et al., 2016; Kobayashi et al., 2018). Stages of phagocytosis start with the formation of the phagosome and then continue with the maturation of the phagosome and finally with the phagolysosome formation (Flannagan et al., 2009).

Myelin is an extension of oligodendrocytes’ plasma membrane in the central nervous system (CNS) (Bunge, 1968; Hildebrand et al., 1993; Simons and Nave, 2015). This extended plasma membrane wraps around axons to create compact myelin sheaths (Demerens et al., 1996; Williamson and Lyons, 2018; Stadelmann et al., 2019). Myelin sheaths enable fast salutatory conduction of action potentials, acting as electrical insulators and allowing for impulse propagation along the axon diameter (Ford et al., 2015; Hughes and Appel, 2016; Williamson and Lyons, 2018). Myelin contains approximately 70% lipids and 30% proteins (Roots et al., 1991; Jackman et al., 2009; Fledrich et al., 2018). Proteolipid protein and myelin basic protein together make up 80% of the myelin protein by weight (Price et al., 1997; Wrathall et al., 1998; Arvanitis et al., 2002). Other important myelin proteins include myelin-associated glycoprotein, NogoA family proteins, oligodendrocyte myelin glycoprotein, and chondroitin sulfate proteoglycans, which are important for neuron regeneration and recovery after SCI (Baldwin and Giger, 2015).

Myelin debris, which is generated from the breakdown of myelin sheaths immediately after SCI, persists in the injury site and contributes to regeneration failure because it contains molecules that strongly inhibit axon regeneration and remyelination (Chen et al., 2000; Filbin, 2003; Kotter et al., 2006; Syed et al., 2016). Moreover, myelin debris is actively involved in inflammatory responses during SCI progression (Jeon et al., 2008; Sun et al., 2010; Wang et al., 2015). Therefore, clearance of myelin debris from the injury site is critical for axon regeneration, remyelination, and inflammation resolution. Infiltrating bone marrow–derived Mϕ (BMDMϕ) and resident microglia are the two major professional phagocytes for myelin debris clearance. Complement-3 receptor (CR3), Mac-2 (Glactin-3), CD36, scavenger receptor AI/II (SRAI/II), and triggering receptor expressed on myeloid cells 2 (TREM2) have been proposed as receptors for myelin debris phagocytosis by Mϕ and microglia (Kuhlmann et al., 2002; Napoli and Neumann, 2010; Sun et al., 2010; Zhou et al., 2014; Wang et al., 2015; Kopper and Gensel, 2018). The semiprofessional phagocytes, such as astrocytes and endothelial cells, can engulf myelin debris as well (Zhou et al., 2019; Konishi et al., 2020; Wang et al., 2020). We recently demonstrated that newly formed microvessels and lining microvascular endothelial cells in the injured spinal cord can engulf IgG-opsonized myelin debris (Zhou et al., 2019).

The role of neutrophils with respect to phagocytosing myelin debris post-SCI is not clear; however, engulfment of myelin debris by neutrophils has been studied in Wallerian degeneration (WD), a degeneration associated with the breakdown of the myelin sheath (Qin et al., 2012; Lindborg et al., 2017). In a mouse model of WD, deletion of neutrophils resulted in a significant lack of myelin debris clearance (Lindborg et al., 2017). Despite the results that neutrophils facilitate compensatory mechanism of clearance of myelin debris in a collaboration with clearance activity of Schwann cells in peripheral nervous system, there is no direct evidence showing that neutrophils are responsible for clearance of myelin debris, and the receptors utilized by neutrophils for myelin debris uptake are as of yet unknown (Lindborg et al., 2017). As a consequence, there are gaps in our knowledge that prevents us from understanding the full functional capacity of neutrophils in SCI in addition to their contribution to secondary tissue damage and recovery post-injury.

### Neutrophil Extracellular Traps

In 2004, it was discovered that neutrophils release extracellular fibers that contained granular proteins and chromatin that trap bacteria (Brinkmann et al., 2004). Most commonly, NETs have been described as a response mechanism to kill extracellular pathogens (Brinkmann et al., 2004; Beiter et al., 2006; Ramos-Kichik et al., 2009; Papayannopoulos et al., 2010; Pilsczek et al., 2010; Juneau et al., 2011; Kaplan and Radic, 2012; Manda et al., 2014; Rosales et al., 2016). It is dependent on the size of the microbe and whether the microbe was able to avoid phagocytosis (Estua-Acosta et al., 2019). The release of NETs is also deadly to the neutrophils themselves and is thus classified as a type of cell death called NETosis, or suicidal NETs (Steinberg and Grinstein, 2007; Papayannopoulos, 2018). NETosis is stimulated by binding molecules to the receptors on the neutrophils such as Toll-like receptors (TLRs), FcRs, and CRs (Brinkmann et al., 2004; Munks et al., 2010; Garcia-Romo et al., 2011; Kaplan and Radic, 2012). Neutrophils about to undergo NETosis display distinctive morphological features that differ from their natural appearance (Fuchs et al., 2007; Vorobjeva and Pinegin, 2014; Scieszka et al., 2020).

Molecules that can stimulate NETs, referred to as sterile stimuli, include cytokines, DNA/RNA and histones, crystals, autoantibodies, and immune complexes (Keshari et al., 2012; Schorn et al., 2012; Behnen et al., 2014; Yalavarthi et al., 2015; Shrestha et al., 2019). NETs are initiated as NADPH is activated following the binding of proinflammatory cytokines, such as TNF-α and TLR-binding molecules (El-Benna et al., 2008; Stoiber et al., 2015). NADPH forms ROS, which can easily be converted to radicals spontaneously or via superoxide dismutase (Bedard and Krause, 2007; Stoiber et al., 2015). An increase in ROS stimulates the release of elastase from the membrane complex of azurophilic granules into the cytosol, activating their proteolytic activity and translocating them into the nucleus, where they aid in chromatin decondensation. Before their translocation into the nucleus, active elastase in the cytosol binds actin and degrades it. This contributes to the plasma membrane and nuclear membrane permeability and the release of NETs into the extracellular space (Metzler et al., 2014; Thiam et al., 2020). NETs are eventually degraded by DNase 1 and cleared by Mϕ (Gupta and Kaplan, 2016). The most important contents of the NETs include but are not limited to (1) granular components: elastase, lactoferrin, azurocidin, cathepsin G, myeloperoxidase (MPO), defensins, and lysozyme; (2) nuclear components: histones H2A, H2B, H3, H4, and myeloid nuclear differentiation agents; (3) cytoplasmic components: S100 calcium-binding proteins A8, A9, and A12; (4) cytoskeletal components: actin, myosin, plastin, and cytokeratin; (5) catalase peroxisome; and (6) glycolytic enzymes enolase and transketolase (Kaplan and Radic, 2012; Vorobjeva and Pinegin, 2014; Estua-Acosta et al., 2019).

There are no current NET formation reports in the injured spinal cord; however, it has been reported that infiltrated neutrophils in CNS release NETs, which may contribute to the blood–brain barrier damage and neural injury in some CNS disorders such as neurodegeneration, multiple sclerosis, traumatic brain injury (TBI), and ischemic stroke (Tillack et al., 2013; Perez-de-Puig et al., 2015; Laridan et al., 2017; Pietronigro et al., 2017; Valles et al., 2017; Ducroux et al., 2018; Farkas et al., 2019; Novotny et al., 2020; Vaibhav et al., 2020). Vaibhav et al. (2020) reported recently that NET formation worsens TBI outcomes, which is regulated by TLR4 and downstream kinase peptidylarginine deiminase 4 (PAD4). Importantly, therapeutically targeting NETs by administration of recombinant human DNase-I degrades NETs and improves neurological outcomes (Vaibhav et al., 2020). As little is known about the mechanisms of NET formation and how it interacts with other CNS resident cells in SCI, it is critical to determine to what degree this neutrophilic defense process partakes in progression of secondary injury in SCI. The research on the clear mechanistic view of NET formation could lead to identifying new targets for therapeutic interventions to treat not only SCI but also other CNS disorders.

### Potentially Detrimental Roles of Infiltrating Neutrophils in SCI

In SCI, neutrophilic MPO activity can be measured within 3 h of the SCI and lasts up to 3 days postinjury. Within 1 day post-SCI, abundant neutrophils can be detected at the injury lesion (Figure 1; Wang et al., 2015). As neutrophils have not been extensively studied in SCI, not much information is available on this topic; however, there are reports regarding the general recruitment of neutrophils to an area of peripheral injury. Infiltration of neutrophils to the injury site has mainly been described as a negative event due to exposure of the injured area to the neutrophilic tissue-damaging factors. Of the four different granules in the neutrophils (Table 3), the most toxic are azurophilic, which cause tissue damage (Lacy, 2006; Nguyen et al., 2007; Kumar and Sharma, 2010). Neutrophils also secrete ROS and proteases such as metalloproteinases (MMPs), contributing to secondary tissue damage (Trivedi et al., 2006). For example, MMP9 facilitates neutrophil penetration of white and gray matter in the injured spinal cord (Fleming et al., 2006). During the first 3 days after injury, there is an increase in MMP9 and NADPH marker gp91^phox^, in the necrotic and apoptotic areas. Both MMP9 and NADPH contribute to the inflammatory response and secondary injury as they generate byproducts such as hydrochlorous acid (HOCl), which cause tissue damage in the surrounding area (Fleming et al., 2006).

A constituent of azurophilic granules of neutrophils is the enzyme elastase, which can create a lot of damage to the surrounding tissues (Doring, 1994; Borregaard and Cowland, 1997; Lominadze et al., 2005). Additionally, elastase can induce cell damage and dysfunction, degrade extracellular matrix proteins, and cause cell death by interfering with normal cellular pathways. Elastase is released into the surrounding tissue after neutrophils degranulate or neutrophils release NETs, resulting in inflammation (Kumar et al., 2018). In chronic inflammation, elastase is present outside neutrophils in high concentrations as elastase inhibitor, α1-proteinase inhibitor, can be easily inactivated by the proteolytic and oxidative attack, but the details of this mechanism remain unknown. In the case of SCI, in response to proinflammatory cytokines, elastase disrupts the neurovascular unit by inducing endothelial cell apoptosis and degrading endothelial cell junction proteins (Kumar et al., 2018). Inhibition of elastase by the administration of sivelestat, an inhibitor of human neutrophil elastase, after an SCI, resulted in the rescue of angiopoietin-1, a vascular growth factor responsible for anti-inflammatory effects and reduction of vessel permeability (Kumar et al., 2018).

MPO is another enzyme of the neutrophils’ azurophilic granules (Borregaard and Cowland, 1997; Lominadze et al., 2005; Kato, 2016). Following the degranulation of neutrophils, MPO is responsible for the generation of various acids, depending on the ions present in the environment, such as HOCl, to kill bacteria (Kato, 2016). MPO has been referred to as the local mediator of tissue damage and inducer of the inflammatory response (Aratani, 2018). It has been reported that MPO worsens secondary injury (Kubota et al., 2012). MPO-KO mice had less HOCl in the injured area, more intact myelin in the core of the injury, and an overall decrease in the production of proinflammatory cytokines such as IL-6, IL-1β, and TNF-α, all of which are contributors to secondary injury of SCI (Hausmann, 2003; Kubota et al., 2012). Locomotor function of MPO-KO mice was significantly better than that of wild-type (WT) mice, indicating an overall increase in functional recovery of MPO-KO injured mice (Kubota et al., 2012). We previously demonstrated that Mϕ that have taken up myelin debris have decreased phagocytic capacity for apoptotic neutrophils (Wang et al., 2015). Nonengulfed apoptotic neutrophils undergo secondary necrosis and release MPO and elastase, which might be involved in inflammation and secondary injury after SCI (Smith, 1999; Wang et al., 2015).

Leukotriene B4 (LB4) is a proinflammatory moderator that induces recruitment of neutrophils through LTB4 receptor 1 (BLT1) on the neutrophils (Crooks and Stockley, 1998). LB4 is produced from arachidonic acid and activated in the lesion area of SCI (Xu et al., 1990). It has been shown that LB4 is involved in the tissue damage caused by neutrophils (Crooks and Stockley, 1998). Neutrophil infiltration in BLT1-KO mice was significantly less than that in WT mice (Saiwai et al., 2010). Coculture of neural cells with neutrophils isolated from the lesion area showed a significant increase in neural cell death compared to neural cells cocultured with circulating neutrophils suggesting that the toxicity present in the lesion area is possibly because of infiltrated neutrophils (Saiwai et al., 2010).

In the earlier section, we discuss neutrophils’ ability to form NETs in response to inflammation and/or infection. Because of the nature of the components that make up the NETs, there is a great potential for tissue damage in this neutrophilic response (Villanueva et al., 2011; Saffarzadeh et al., 2012; Carmona-Rivera et al., 2015; Sorensen and Borregaard, 2016; Brinkmann, 2018). As infiltrated neutrophils are accumulated in lesion core (Figure 1), where the demyelinating area for new blood vessel formation is located, it is likely that NET formation and NETosis may be involved in the pathogenesis of SCI such as destruction of myelin sheath and BSCB breakdown if NETs were to be detected in SCI.

### Potentially Beneficial Roles of Infiltrating Neutrophils in SCI

Although recruitment of neutrophils is thought of as damaging for the injured tissue, there is also a positive aspect of that recruitment. As first responders to the injured site, they can initiate clearance of debris and produce proinflammatory signals that recruit other immune cells such as Mϕ to eliminate leftover debris and contribute to tissue healing (Trivedi et al., 2006). Defective neutrophil recruitment to the injury site, mediated by the absence in the expression of esophageal cancer–related gene 4 (ECRG4) causing suppression of CD44, shows impaired wound healing process (Dorschner et al., 2020). A study reported that depletion of Ly6G/Gr-1 neutrophils in SCI mice resulted in the abolition of neutrophil infiltration into the injured spinal cord, lack of appropriate proinflammatory response, and impairment of injury recovery (Stirling et al., 2009).

Neutrophils are a heterogeneous cell population essential for immune defense versatile in their defense mechanisms. Heterogeneity of neutrophils is defined by the maturity of the cells, activation state, and discrete subsets such as low-density neutrophils, immunomodulatory neutrophils, and neutrophils expressing surface maker CD177 (Grieshaber-Bouyer and Nigrovic, 2019; Ng et al., 2019). A recent study reports a new subset of neutrophils with axon regenerative properties (Sas et al., 2020). This subset is characterized as CD14^+^Ly6G^lo^ granulocytes resembling immature neutrophils. In SCI, this new subset promoted axonal regrowth, passing beyond the injury site and exhibiting neuroprotective and proregenerative functions (Sas et al., 2020). Therefore, heterogeneity of neutrophils offers new therapeutic opportunities.

The secretory leukocyte protease inhibitor (SLPI) is a serine protease inhibitor and a member of the innate immune system with an anti-inflammatory role (Doumas et al., 2005). It protects the tissue from damage by forming protease–antiprotease complexes (Ying et al., 1994). SLPI is expressed by neutrophils and is an important factor in SCI recovery and the improvement of locomotor activity by pausing further degradation of tissue matrix and thereby preventing further secondary damage (Ghasemlou et al., 2010). Recombinant SLPI treatment in SCI mice showed a reduced lesion size in the epicenter of the injury and reduced myelin loss, indicating a lesser degree of secondary injury (Ghasemlou et al., 2010). Overexpression of SLPI inhibited proinflammatory signals and significantly improved locomotor activity (Ghasemlou et al., 2010; Svensson et al., 2017).

## Conclusion

Although we started to understand the events in secondary injury better, we have yet to uncover the early contributors of secondary injury and why secondary injury has such irreversible consequences. Neutrophils are the first immune cells to infiltrate the injured spinal cord (Popovich and Jones, 2003; Oyinbo, 2011; Kubota et al., 2012; Guo et al., 2016). The role of neutrophils in innate immunity has been well studied, their response to the pathogenic invasion is well characterized, and their infiltration into the injured tissue has been thoroughly reported. Nevertheless, their role in SCI and their contribution to the secondary injury cascade in SCI are not well understood. The roles of neutrophils in SCI may be context-dependent. We understand that neutrophils are important in propagation of the inflammatory response and recruitment of other immune cells, such as BMDMϕ to the injured area for clearance of damaged cells and cellular debris (Prame Kumar et al., 2018). However, it remains unclear what are the other cellular functions of neutrophils in the injured area. Understanding the role of neutrophils at the different stages of SCI could lead to major advances in determining the timeline of the irreversible and detrimental changes following an SCI and allow us to pinpoint the best therapeutic target.

## Author Contributions

SZ contributed to data collection, critical analysis of the literature, and writing the manuscript. MA and GH contributed to the discussion. YR contributed to the conception, design, critical analysis of the literature, and writing the manuscript. All authors contributed to the article and approved the submitted version.

## Conflict of Interest

The authors declare that the research was conducted in the absence of any commercial or financial relationships that could be construed as a potential conflict of interest.
